# Leukocyte mRNA sequencing reveals unfolded protein response activation in severe heat stroke in Japan

**DOI:** 10.3389/fcell.2025.1640477

**Published:** 2025-11-27

**Authors:** Yusuke Nomura, Sayaka Oda, Hisatake Matsumoto, Yuki Togami, Junya Shimazaki, Daisuke Okuzaki, Hiroshi Ogura, Jun Oda

**Affiliations:** 1 Yokohama City University Hospital, Yokohama, Kanagawa, Japan; 2 Laboratory for Human Immunology (Single Cell Genomics), Immunology Frontier Research Center, Suita, The University of Osaka, Osaka, Japan; 3 Department of Traumatology and Acute Critical Medicine, The University of Osaka Graduate School of Medicine, Suita, Osaka, Japan; 4 Department of Acute Medicine and Critical Care Medical Center, National Hospital Organization Osaka National Hospital, Osaka, Japan

**Keywords:** severe heat stroke, whole blood transcriptome, mRNA, intensive care, UPR pathway

## Abstract

**Background:**

Severe heat stroke can cause multi-organ dysfunction and can be fatal, yet its underlying pathophysiology remains poorly understood. While RNA alterations are known to occur in critical illness, their specific role in severe heat stroke has not been comprehensively studied. Understanding these molecular changes may improve diagnostic and therapeutic strategies. This study aimed to investigate the pathogenesis of severe heat stroke by analyzing RNA expression profiles in Japanese patients in comparison to healthy controls.

**Results:**

This single-center, prospective, observational study included seven patients (median age: 69 ± 21.5 years; 57.1% male) with severe heat stroke admitted to Osaka University Hospital Emergency Center between August 2020 and July 2021, along with five healthy controls. All patients exhibited central nervous system symptoms and renal dysfunction; 28.6% had hepatic dysfunction or coagulopathy, and one patient (14.3%) died. RNA sequencing of leukocyte total RNA identified 15,876 RNAs, among which 1,106 were upregulated and 1,000 were downregulated in patients relative to controls (|log2 fold change| > 1, false discovery rate < 0.1). Pathway analysis revealed significant activation of the endoplasmic reticulum stress response, particularly the unfolded protein response, which is involved in cellular stress adaptation and survival mechanisms.

**Conclusion:**

Severe heat stroke in Japanese patients was associated with widespread changes in RNA expression, highlighting activation of the endoplasmic reticulum stress response. These findings provide new insights into the pathophysiology of heat stroke and suggest that targeting cellular stress pathways may be a potential therapeutic approach. Further research is needed to validate these results in larger cohorts and assess their clinical implications.

## Background

Organ damage from severe heat stroke can affect multiple systems, such as the central nervous system, liver, kidneys, and the coagulation cascade, leading to potentially fatal outcomes. It is reported that 4.6% of heat stroke patients admitted to hospitals in Japan die, with one-sixth of them exhibiting poor neurological outcomes (Shimazaki et al., 2020). The pathophysiology is believed to involve a coordinated stress response mediated by heat shock protein chaperone families, as well as inflammatory and anti-inflammatory cytokines, which affect endothelial cells, leukocytes, and epithelial cells. Nevertheless, the detailed molecular mechanisms underlying these responses in heat stroke remain to be fully elucidated (Yang and Lin, 1999; Yan et al., 2006; Dehbi et al., 2010).

With advances in molecular biology, it is now possible to uncover new disease mechanisms by comprehensively analyzing gene expression and protein synthesis. Alterations in RNA profiles have been observed under various critical conditions, and we have previously documented changes in mRNA associated with COVID-19 (Togami et al., 2022), acute respiratory distress syndrome (Mitsuyama et al., 2024), sepsis (Muratsu et al., 2024; Oda et al., 2025), and methemoglobinemia (Mitsuhara et al., 2023). There is also previous research on patients with severe heat stroke using whole genome microarray analysis (Bouchama et al., 2023). RNA-Seq is more sensitive in detecting genes with very low expression and more accurate in detecting the expression of extremely abundant genes (Zhao et al., 2014). RNA-Seq also has a wider dynamic range than microarray analysis (Zhao et al., 2014). Therefore, such RNA profiling from microarray analysis has yet to be extensively explored in the context of severe heat stroke. This study aimed to compare the mRNA levels in whole blood samples from patients with severe heat stroke admitted to the intensive care unit to those of healthy individuals. By identifying the expression of unique RNAs associated with severe heat stroke, we aim to enhance our understanding of its molecular basis. Such insights could pave the way for the development of novel therapeutic and preventive strategies against this life-threatening condition.

## Materials and methods

### Study design and participants

This single-center, prospective, observational study was conducted at The University of Osaka Hospital. Among 1,131 patients admitted to the intensive care unit between August 2020 and July 2021, eight were diagnosed as having heat stroke. Of these, seven patients were included in the present study because their blood samples were collected within 24 h of admission. Diagnosis in all patients was based on an elevated core body temperature exceeding 40 °C and central nervous system dysfunction, such as delirium, convulsions, or coma (Bouchama and Knochel, 2002), and other diagnoses were excluded. To elucidate the pathogenesis of heat stroke, mRNA in the whole blood of these patients was analyzed.

### Clinical data

Clinical data collected from each patient’s electronic medical records by the investigators included age, sex, body mass index, Acute Physiology and Chronic Health Evaluation II and Sequential Organ Failure Assessment scores, comorbid conditions such as hypertension, diabetes, and hyperlipidemia, and clinical variables such as the hospital outcome.

### Identification of mRNA expression, library preparation, and RNA sequencing

Total RNA isolation was performed using the PAXgene Blood RNA System (BD Bioscience, San Jose, CA, United States) from blood drawn from seven patients with severe heat stroke and five healthy subjects as controls. All blood samples for the analyses were collected in collection tubes and stored at −30 °C until further analysis. Library preparation was performed according to the manufacturer’s instructions using a TruSeq stranded mRNA sample prep kit (Illumina, San Diego, CA). These libraries were converted to libraries for DNBSEQ using an MGIEasy Universal Library Conversion Kit (App-A). Sequencing was performed on a DNBSEQ-G400 platform in 2 × 100 bp paired-end mode. During the sequencing run, control software automatically operates base calling analysis software and delivers raw sequencing data outputs. The sequenced reads were then mapped to the human reference genome sequences (hg19) using TopHat (ver. 2.1.1) (Kim et al., 2013). The fragments per kilobase of exons per million mapped fragments were calculated using Cuffdiff (ver. 2.2.1) (Trapnell et al., 2012).

### Statistical analysis of mRNA

The raw counts were analyzed using the edgeR package (Robinson et al., 2010). Multi-dimensional scaling with the “cmdscale” command in R (Oda et al., 2025) was performed to compare gene expression between the control subjects and the patients with severe heat stroke. Volcano plot analysis was conducted to visualize and identify the significant changes in the expression lists. Significance was defined by |log2 fold change| > 1 and false discovery rate (FDR) < 0.1. mRNAs with significantly different expression levels were subjected to further analysis (Goedhart and Luijsterburg, 2020). To evaluate functional characteristics and upstream regulators of RNA expression levels, the data were analyzed by canonical signaling pathway analysis and upstream regulator analysis with calculation of z-scores and *P* values in Ingenuity Pathway Analysis (IPA) (QIAGEN Inc.). The z-score predicts the activation state of the upstream regulator using RNA expression patterns of the downstream state of that regulator. The canonical pathway and upstream regulator were considered activated if the |z-score| was > 2 with *P* < 0.05. The canonical pathway analysis used significantly different mRNA expression levels to describe specific relations between RNAs. Gene Ontology (GO) enrichment analysis of the analyzed RNAs was conducted using the clusterProfiler package in R (Yu et al., 2012).

## Results

### Patient characteristics

Seven patients with severe heat stroke and five control subjects were analyzed. The median age of the patients was 69 ± 21.5 years, and 4 (57.1%) patients were male. All patients had central nervous system symptoms and renal dysfunction, two patients (28.6%) had hepatic dysfunction, and two patients (28.6%) had coagulopathy. One patient (14.3%) died. Details of the patients’ characteristics are shown in Table 1. There were no significant differences between the patients and healthy control subjects.

**TABLE 1:** Patient characteristics.

Characteristic	Controls	Heat stroke
	(N = 5)	(N = 7)
Age, years, median, IQR	59 (64–83)	69 (47.5–80)
Sex, male (%)	3 (60)	4 (57.1)
BMI, kg/m^2^, median, IQR	21.3 (19.1–24.9)	20.6 (17.9–21.3)
Comorbidities, n (%)		
Diabetes	2 (40)	1 (14.3)
Hypertension	2 (40)	0 (0)
Hyperlipidemia	1 (20)	0 (0)
Hyperuricemia	1 (20)	0 (0)
Chronic heart disease	1 (20)	0 (0)
Chronic lung disease	0 (0)	0 (0)
Chronic kidney disease	0 (0)	0 (0)
Immunocompromised condition	0 (0)	0 (0)
Malignant neoplasm	0 (0)	0 (0)
In-hospital mortality		1 (14.3)
SOFA score, median, IQR		9 (7.5–11.5)
APACHE II score, median, IQR		29 (29–32)
Intubation required, n (%)		6 (85.7)
Core temperature (°C)		40.5 (40.3–41.4)
Symptoms, n (%)		
CNS dysfunction		7 (100)
Renal dysfunction		7 (100)
Hepatic dysfunction		2 (28.6)
Coagulopathy		2 (28.6)

IQR, interquartile range; BMI, body mass index; SOFA, sequential organ failure assessment; APACHE, acute physiology and chronic health evaluation; CNS, central nervous system. Data are shown as group number (percentage) or median (interquartile range).

### RNA expression and multidimensional scaling

**FIGURE 1:**
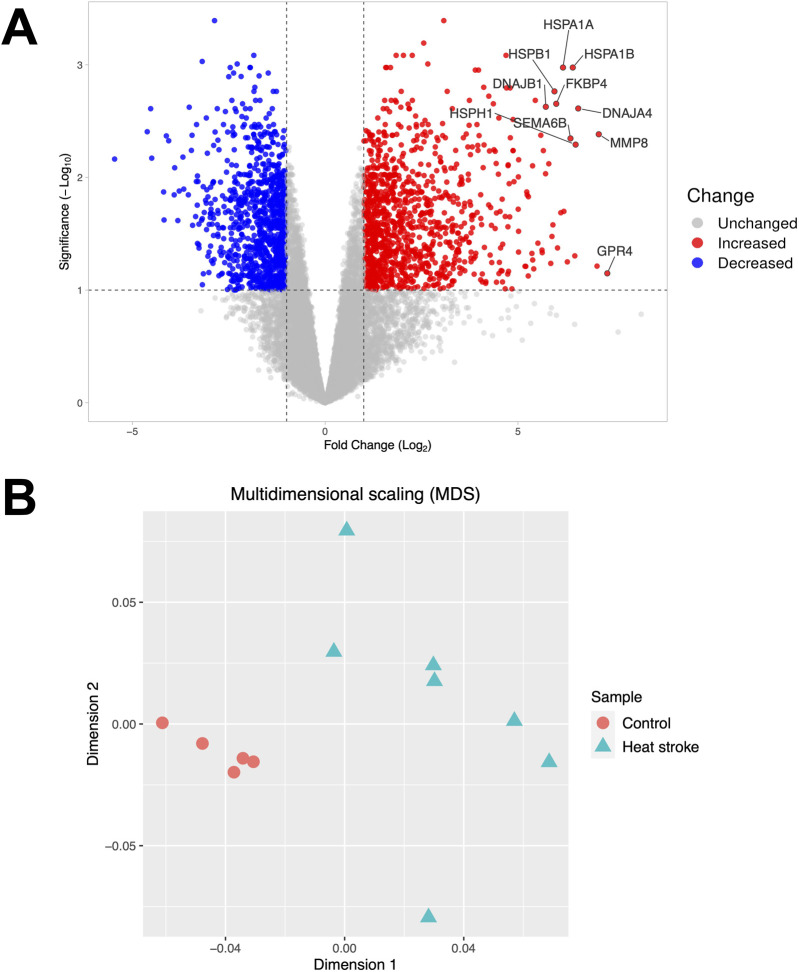
Volcano plot and multidimensional scaling. **(A)** Volcano plot representing the expression of differentially expressed mRNAs in severe heat stroke compared to the control subjects. Among the differentially expressed RNAs, 1,106 mRNAs were upregulated, and 1,000 mRNAs were downregulated. The significant differentially expressed RNAs are indicated. The vertical dotted lines represent |log2 fold change| > 1. The horizontal dotted line represents the threshold for a false discovery rate < 0.1. Red dots indicate upregulated RNAs, and blue dots indicate downregulated RNAs. **(B)** Multidimensional scaling plot of the mRNA expression in severe heat stroke compared to the control subjects. Each dot indicates a patient or control.

Of 15,876 RNAs detected, 1,106 were upregulated, and 1,000 were downregulated (|log2 fold change| > 1, FDR < 0.1) relative to the control subjects (Figure 1A). Multidimensional scaling showed that mRNA expression levels could be used to distinguish between the heat stroke patients and healthy control subjects (Figure 1B).

### Canonical pathway, upstream regulators, and GO enrichment analyses

**FIGURE 2:**
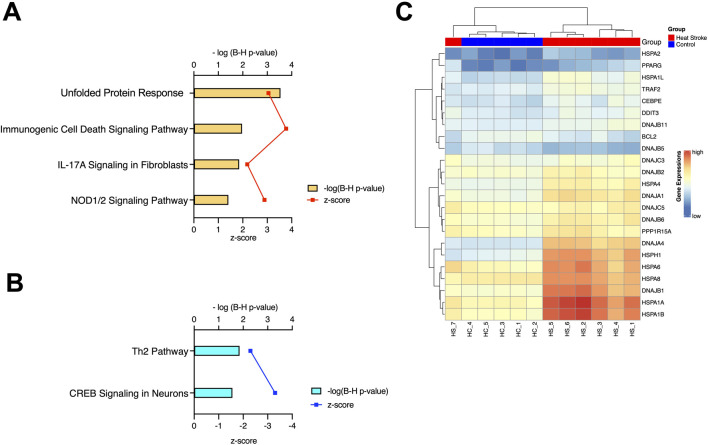
Canonical pathway analysis. **(A)** Activated canonical pathways in severe heat stroke mRNA identified using Ingenuity Pathway Analysis. **(B)** Inhibited canonical pathways in severe heat stroke mRNA identified using Ingenuity Pathway Analysis. **(C)** Heatmap of the expression of genes involved in the unfolded protein response pathway of the samples, as calculated through RNA-Seq. B-H, Benjamini-Hochberg; IL, interleukin; NOD, nucleotide-binding oligomerization domain; CREB, cyclic AMP response element-binding protein.

**FIGURE 3:**
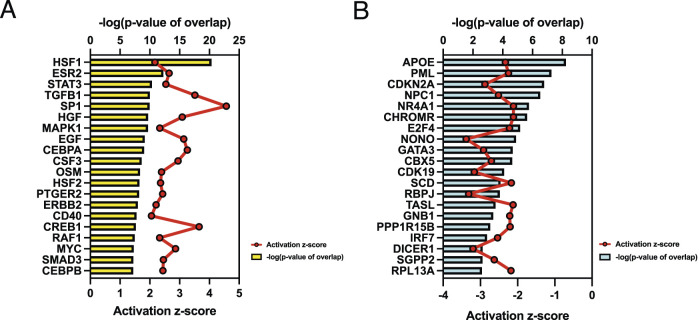
Upstream regulator analysis. **(A)** Top 20 activated upstream regulators. **(B)** Top 20 inhibited upstream regulators.

**FIGURE 4:**
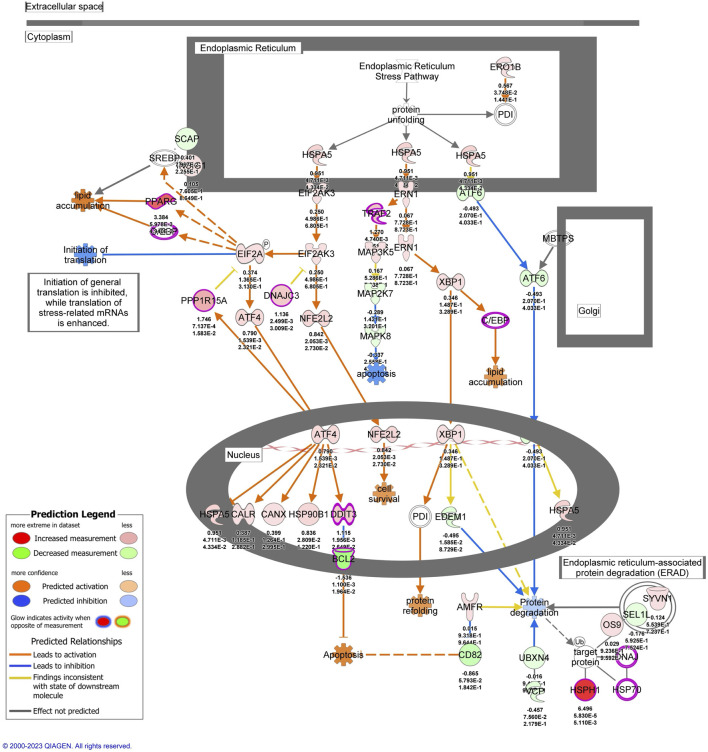
The activated unfolded protein response pathway predicted by Ingenuity Pathway Analysis. mRNAs with |log2 fold change| > 1 and false discovery rate < 0.1 were included in the pathway.

To investigate signaling pathways significantly involved in severe heat stroke, outcomes from RNA sequencing were subjected to canonical pathway analysis in IPA (2023 Summer), and the activated canonical signaling pathways in severe heat stroke were screened and listed. Canonical pathway analysis predicted the activation of four pathways (Figure 2A) and the inhibition of two pathways (Figure 2B). The unfolded protein response (UPR) pathway had the highest Benjamini-Hochberg-adjusted *P* value of 2.9E-4 with z-score = 3.051. Twenty-three mRNAs were involved in the UPR pathway (Figure 2C). Analysis of upstream regulators identified 341 activated potential upstream regulators and 155 inhibited potential upstream regulators (*P* value of overlap < 0.05). Each of the top 20 regulators is shown in Figures 3A,B, respectively. Canonical signaling pathway analysis in IPA showed the predicted relations between RNAs in the activated UPR pathway (Figure 4). The results of the GO enrichment analysis (related to biological processes) showed that the protein folding pathway (enrichment FDR = 2.3E-10, fold enrichment = 4.3), chaperone-mediated protein folding pathway (enrichment FDR = 6.3E-9, fold enrichment = 6.3), and response to unfolded protein pathway (enrichment FDR = 4.3E-6, fold enrichment = 4.0) were significant for both FDR and fold enrichment (shown in Supplementary Figure S1). The results of the Kyoto Encyclopedia of Genes and Genomes (KEGG) pathway analysis are shown in Supplementary Figure S2.

## Discussion

Heat stroke leads to the degradation and aggregation of numerous intracellular proteins, particularly those involved in nuclear structure and the cytoskeleton (Yan et al., 2006). Previous studies have shown significant alterations in nuclear integrity, often accompanied by inhibition of ribosomal formation (Hildebrandt, 2002; Yan et al., 2006). This is primarily due to heat-induced protein misfolding, which increases the concentration of nuclear proteins, causing intracellular protein insolubility (Yan et al., 2006).

Our analysis of whole blood RNA samples from the seven patients with severe heat stroke revealed upregulation of 1,106 RNAs and downregulation of 1,000 RNAs relative to samples from the five control individuals. These RNA expression trends differed significantly between the two groups, indicating alterations in RNA profiles in the patients with severe heat stroke. Further analysis of the upregulated RNAs indicated significant activation of the UPR pathway. The endoplasmic reticulum (ER) extends throughout the cytoplasm and is continuous with the nuclear envelope, enabling it to sense and transmit signals originating from various cellular compartments. The ER has evolved as a specialized organelle for protein folding that facilitates proper folding and prevents aggregation. When misfolded or unfolded proteins accumulate within the ER, the UPR, a conserved cellular stress mechanism, is activated. The UPR functions to restore ER homeostasis by halting protein synthesis, promoting the degradation of misfolded proteins, and upregulating molecular chaperones involved in protein folding (Hildebrandt, 2002; Kaufman et al., 2002; Schröder and Kaufman, 2005; Hetz, 2012).

If these adaptive mechanisms fail to relieve stress within a certain timeframe, or if ER stress persists, the response can shift toward cell death (Kaufman et al., 2002; Schröder and Kaufman, 2005). In summary, the UPR initially acts as a cytoprotective mechanism that restores normal cell function under manageable stress. However, when stress is prolonged or severe, the UPR may adopt a cytotoxic role, promoting cell death through apoptotic pathways. In severe heat stroke, during which cellular stress is intense and sustained, the UPR may predominantly function in this cytotoxic manner, thus contributing to cellular injury and dysfunction. This shift underscores the dual nature of the UPR, with outcomes dependent on the duration and magnitude of the stressor (Kaufman et al., 2002; Schröder and Kaufman, 2005).

The UPR involves three signaling pathways: IRE1, PERK, and ATF6. It serves as a central signaling mechanism that determines cell fate under ER stress by regulating whether the cell undergoes apoptosis or survives. The UPR has essential functions in many physiological processes, including lipid and energy metabolism, innate immunity, and cell differentiation (Hildebrandt, 2002).

Bouchama et al. (2023) examined the whole genome transcriptome in peripheral blood mononuclear cells of a cohort of subjects exposed to the same high environmental heat conditions with or without the development of heat stroke. The transcriptome showed that genes involved in more than half of the entire chaperone were differentially expressed relative to heat stress controls. These included the heat shock protein, co-chaperone, and chaperonin genes, which indicated a robust heat shock response. Differentially expressed genes also encoded proteins related to the UPR, DNA repair, energy metabolism, oxidative stress, and immunity (Bouchama et al., 2023). Compared with microarray analysis, RNA-Seq can detect novel transcripts and isoforms, map exon/intron boundaries, discover sequence variations, and reveal splice variants (Zhao et al., 2014). For the study of differential gene expression, RNA-Seq does not suffer from hybridization-based limitations associated with microarray analysis (e.g., background noise and saturation) or with probe set issues (e.g., incorrect annotation and isoform coverage) (Zhao et al., 2014). Our study showed similar results and predicted perturbations of the proteome network and energy production.

## Limitations

This study has several limitations. First, it included a limited number of patients with severe heat stroke, and the results need to be validated in larger prospective cohorts. Second, clinical backgrounds such as pre-existing conditions, treatments before admission, and comorbidities were not fully evaluated. Third, the study design did not include individuals exposed to environmental heat who did not develop heat stroke. Future investigations using single-cell sequencing or other advanced molecular approaches will be valuable to confirm and expand these findings. Despite these limitations, this study provides important insights into the molecular mechanisms underlying severe heat stroke.

## Conclusion

Compared to healthy control subjects, patients with severe heat stroke showed altered expression of RNAs, especially those involved in activation of the UPR pathway, which is a cellular stress response related to ER stress. This suggests that when cellular stress is intense and sustained, the UPR may primarily function in a biotoxic manner that contributes to cellular injury and dysfunction in cases of severe heat stroke. These findings provide new insights into heat stroke pathophysiology and suggest that targeting cellular stress pathways may be a new approach with therapeutic potential.

## Data Availability

The datasets presented in this study can be found in online repositories. The names of the repository/repositories and accession number(s) can be found below: https://www.ncbi.nlm.nih.gov/geo/, GSE 256062.

## Supplementary material

SUPPLEMENTARY FIGURE S1 Results of Gene Ontology (GO) enrichment analysis for genes related to biological processes. The results showed that the protein folding pathway (enrichment FDR = 2.3E-10, fold enrichment = 4.3), chaperone-mediated protein folding pathway (enrichment FDR = 6.3E-9, fold enrichment = 6.3), and response to unfolded protein pathway (enrichment FDR = 4.3E-6, fold enrichment = 4.0) were significant for both FDR and fold enrichment.

SUPPLEMENTARY FIGURE S2 Results of the Kyoto Encyclopedia of Genes and Genomes (KEGG) pathway analysis.
