# Supplementary figures and images for: Leukocyte mRNA sequencing reveals unfolded protein response activation in severe heat stroke in Japan
Source: Front Cell Dev Biol. 2025 Nov 27;13:1640477. doi: 10.3389/fcell.2025.1640477 (PMC12695859; doi:10.3389/fcell.2025.1640477)

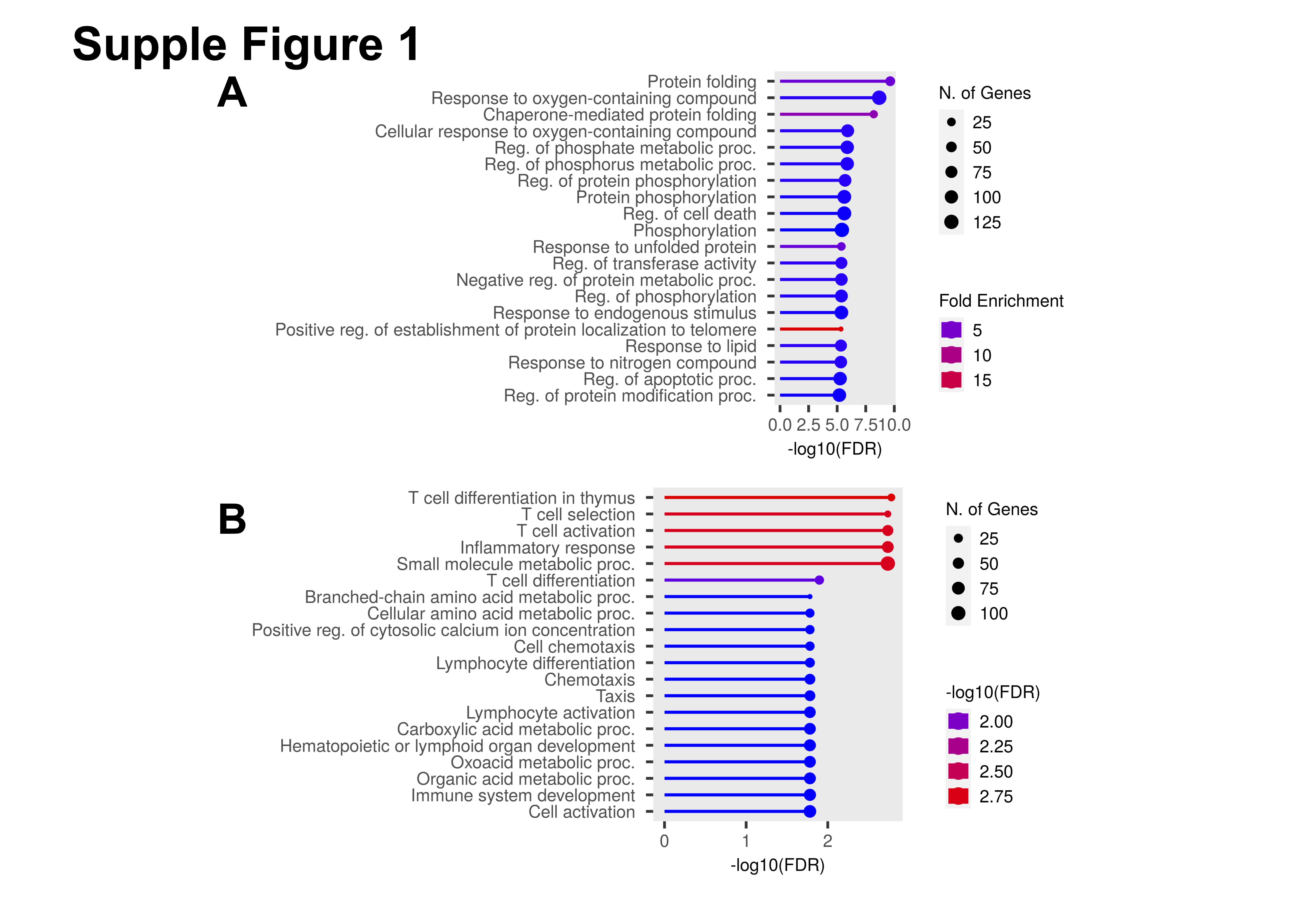

Supplement: Supplementary file 1 [file Image1.jpeg]

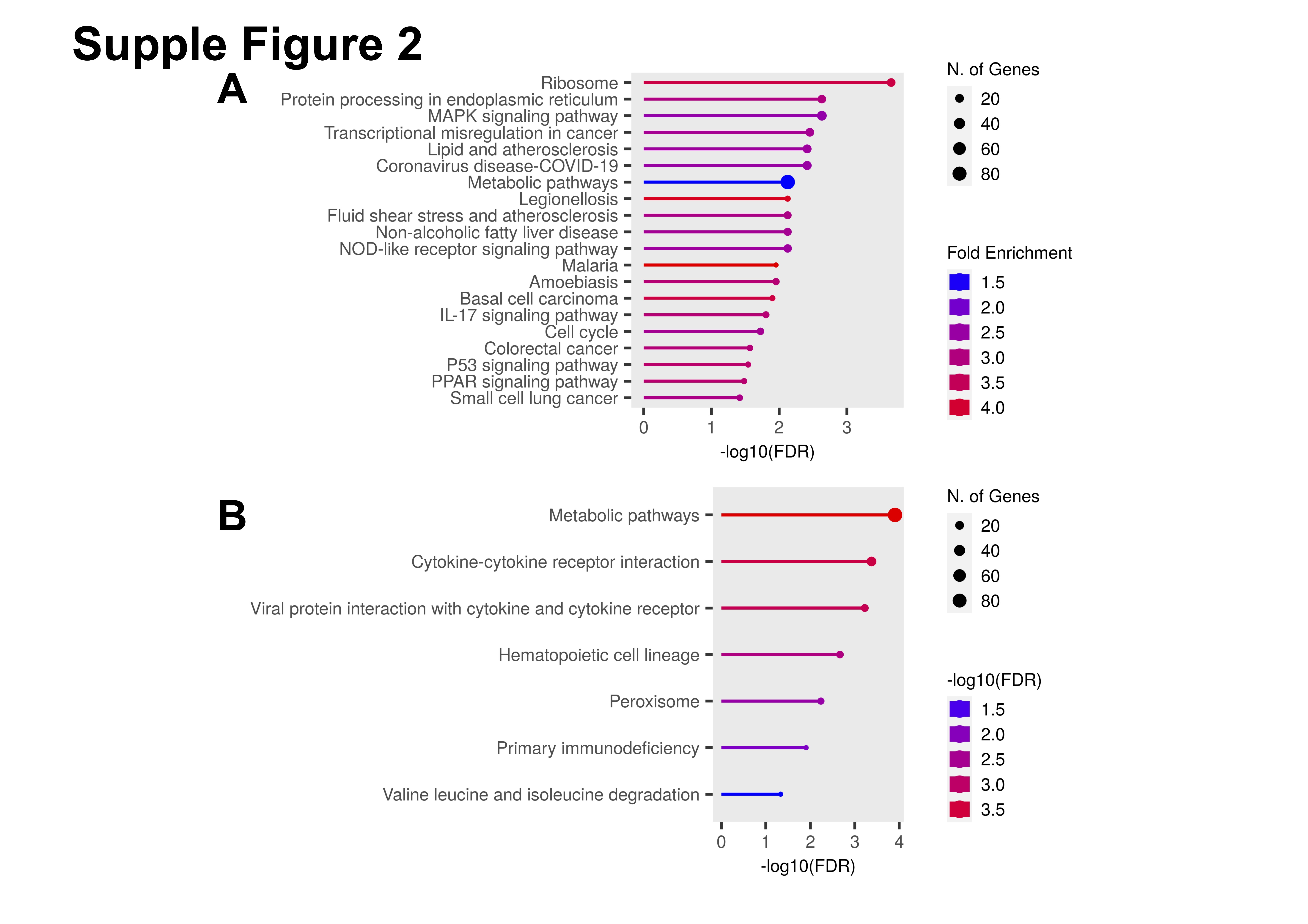

Supplement: Supplementary file 2 [file Image2.jpeg]
